# Editorial: Women in biofilms 2021

**DOI:** 10.3389/fcimb.2022.1035280

**Published:** 2022-10-28

**Authors:** Carina Almeida, Lauren O. Bakaletz

**Affiliations:** ^1^ INIAV - National Institute for Agrarian and Veterinary Research, Food Safety Unit, Vila do Conde, Portugal; ^2^ LEPABE - Laboratory for Process Engineering, Environment, Biotechnology and Energy, Faculty of Engineering, University of Porto, Porto, Portugal; ^3^ Centre of Biological Engineering, Universidade do Minho, Campus de Gualtar, Braga, Portugal; ^4^ Abigail Wexner Research Institute, Center for Microbial Pathogenesis, Nationwide Children’s Hospital, Columbus, OH, United States; ^5^ College of Medicine, Department of Pediatrics, The Ohio State University, Columbus, OH, United States

**Keywords:** women in science, biofilms, biofilm control strategies, biofilm formation, clinical biofilms

Female scientists lead ground-breaking research across the world, however, to date women represent only 33% of researchers worldwide. For instance in Europe, only 11% of senior research roles are held by women (UNESCO, 2021). Female representation decreases with the level of seniority, and this vertical segregation can be encountered in almost every country. A recent study evaluated articles in the medical field and found that those written by women in high-impact medical journals had fewer citations than those written by men, particularly when women wrote together as primary and senior authors (Chatterjee and Werner, 2021). This data clearly demonstrates that long-standing biases and gender stereotypes may have important consequences for the professional success of women and for achieving gender equity in science-related fields. This collection of innovative works can help empower more female scientists to achieve recognition for their scientific work, while participating equally in solving the major challenges in the field of microbial biofilms.

Biofilms are complex three-dimensional structures, corresponding to the sessile mode of microbial life (Flemming et al., 2021). These structures have been progressively recognized as the predominant form of life for microbes on Earth. In this sessile form, microorganisms live in close proximity to one another within a matrix of extracellular polymeric substances (EPS), providing an extra layer of protection against external and/or environmental stresses (Karygianni et al., 2020). In addition to EPS production, these sessile populations usually exhibit slow growth rates and high tolerances to antibiotics, chemical treatments, and host-immune effectors. Collectively, these properties make biofilm control a challenge that has yet to be tackled.

Women play a prominent role in the field of microbial biofilms, taking the lead on several innovative works that contribute to our understanding of these complex and diverse structures and set the basis for important findings. The works presented in this Research Topic boast women as the first or last authors, which indicates their prominent role in leading this research. In particular, this collection advances our understating of biofilm dynamics and properties, effective control strategies, and even biofilm association with diseases previously thought to be unrelated to these structures.

Beginning with a study on biofilm formation mechanisms, Leggett et al. apply a metabolomics approach to biofilm-forming *Pseudomonas aeruginosa* in order to identify factors that trigger the transition from free/planktonic cells to adherence and static growth. The authors demonstrate that cadaverine can decrease biofilm accumulation; this information can eventually be used to design new interventional strategies. Moving on to the virulence traits of biofilms, Fleming et al. explore the contribution of two specific EPS in *P. aeruginosa* pathogenesis based on a wound infection model. The data suggest that the EPS do not affect the severity of wound infection in terms of bacteria load and wound closure rates; however, they may have important implications for bacterial persistence. The metabolic state is also particularly relevant and is addressed in an interesting study by Gaio et al.. The authors explore the relevance of the viable but non-culturable (VBNC) cells in *Staphylococcus epidermidis*, grown in either planktonic or biofilm conditions. Despite the identification of genes possibly associated with the VBNC state, there is no significant difference between the proportion of VBNC cells in sessile or biofilm populations. In a similar way, the same research group attempts to decipher the role played by a putative toxin-antitoxin system in the virulence traits of *S. epidermidis* (Gaio et al.). Despite the previously identified potential role of this system, the results suggest a minor role played in the different virulence factors evaluated. While not all hypotheses are verified, other have been corroborated by resorting to large-scale studies involving a high number of isolates. Thoming and Haussler confirm the high tolerance of *P. aeruginosa* biofilms to antimicrobials in a large study involving 352 clinical isolates. A broad distribution of biofilm-induced tolerance phenotypes is observed; however, these phenotypes are also affected by the antibiotic utilized.

Regarding the development of pioneering assessment strategies, this collection includes an important contribution by Gloag et al. The authors present an innovative method, originally applied to quantify marine biofilm fouling, to accurately detect and quantify the presence and/or detachment of biofilms as a function of shear stress. This rotating-disc rheometry method is used to investigate the effect of arginine on dental plaque-biofilms, corroborating previous observations on its ability to disrupt *Streptococcus gordonii* biofilms.

Regarding control strategies, natural compounds exhibiting anti-biofilm properties can be extremely valuable. The study by Priya et al. provides a good example of bioactive compounds, namely, piperine and thymol (present in pepper and thyme, respectively) that, when combined, display a strong synergistic effect against *Candida albicans* biofilms. This combination of therapies is frequently regarded as an excellent way to overcome biofilms’ tolerance to antimicrobials. As such, Hawas et al. provide a comprehensive overview of combination therapies, primarily based on biofilm dispersing agents and antibiotics, highlighting various synergistic treatments that show outstanding potential.

Finally, exciting findings related to human diseases with little (or unknown) connection to biofilms are also presented. Mateus et al. explore the pathogenesis of pediatric obstructive sleep disordered breathing (SDB) and its potential link to biofilms associated with recurrent tonsillitis. The results suggest that persistent bacterial infection may be related to SDB since the microbial phenotypes are remarkably similar. Thus, the authors argue that anti-biofilm approaches may be relevant in the treatment of SDB. In the same way, Grando et al. explore the role of amyloid proteins; bacterial fibers that are a common component of bacterial biofilm matrixes, in the development of autoimmune diseases. A hypothesis that rests on the fact that autoimmune reactions are often developed after an infectious disease episode and that amyloid proteins have proved to be able to activate a pro-inflammatory response. The results obtained from a mouse model that is implanted with mesh-associated *S. aureus* biofilms (either producing, or defective in the production of, amyloids) provide compelling evidence for the involvement of these amyloid proteins in infection-associated autoimmunity. Last but not least, Ragan et al. describe the use of a probiotic strain, in its biofilm state, to modulate the human gut, preventing gut infections and preserving the natural gut functions.

These works clearly open new avenues in the study and understanding of biofilm structures. They demonstrate how these communities can be modulated not only to control biofilm-associated problems and infections, but also to improve health conditions that are not directly associated with biofilms. This collection of articles showcases women’s contribution to this field of knowledge. Many more examples could be presented here to highlight the diversity of research performed by women across the full breadth of biofilm research.

## Author contributions

CA and LB: drafting and critical revision of the manuscript. Both authors contributed to the article and approved the submitted version.

## Conflict of interest

The authors declare that the research was conducted in the absence of any commercial or financial relationships that could be construed as a potential conflict of interest.

## Publisher’s note

All claims expressed in this article are solely those of the authors and do not necessarily represent those of their affiliated organizations, or those of the publisher, the editors and the reviewers. Any product that may be evaluated in this article, or claim that may be made by its manufacturer, is not guaranteed or endorsed by the publisher.
